# ﻿New *Aquilariomyces* and *Mangifericomes* species (Pleosporales, Ascomycota) from *Aquilaria* spp. in China

**DOI:** 10.3897/mycokeys.112.139831

**Published:** 2025-01-13

**Authors:** Tian-Ye Du, Samantha C. Karunarathna, Kevin D. Hyde, Somrudee Nilthong, Ausana Mapook, Dong-Qin Dai, Kunhiraman C. Rajeshkumar, Abdallah M. Elgorban, Li-Su Han, Hao-Han Wang, Saowaluck Tibpromma

**Affiliations:** 1 Research Center of Natural History and Culture, Center for Yunnan Plateau Biological Resources Protection and Utilization, Key Laboratory of Yunnan Provincial Department of Education of the Deep-Time Evolution on Biodiversity from the Origin of the Pearl River, College of Biology and Food Engineering, Qujing Normal University, Qujing 655011, China; 2 Center of Excellence in Fungal Research, Mae Fah Luang University, Chiang Rai 57100, Thailand; 3 School of Science, Mae Fah Luang University, Chiang Rai 57100, Thailand; 4 National Fungal Culture Collection of India (NFCCI), Biodiversity and Palaeobiology (Fungi) Gr, MACS Agharkar Research Institute, G. G. Agarkar Road, Pune 411004, India; 5 Department of Botany and Microbiology, College of Science, King Saud University, Riyadh 11451, Saudi Arabia

**Keywords:** 2 new species, agarwood, saprobes, Thymeleaceae, Thyridariaceae

## Abstract

Saprobic fungi are known for their critical role in decomposition and nutrient cycling. The study of saprobic fungi is equally important, as it helps in understanding their ecological roles and identifying their hidden diversity. This study focused on saprobic fungi on *Aquilaria*, which is poorly studied compared to economically important hosts like coffee, tea, and rubber. Our rigorous process led to the collection of two new terrestrial saprobic fungi from the Guangdong and Yunnan provinces in China. After extensive phylogenetic analyses and detailed comparison of morphological characteristics, the two collections were identified as two new species belonging to Pleosporales, Ascomycota. *Aquilariomycesmaomingensis***sp. nov.** was isolated from *Aquilariasinensis* in Guangdong Province, while *Mangifericomesaquilariae***sp. nov.** was isolated from *Aquilaria* sp. in Yunnan Province. Full descriptions, photo plates, and phylogenetic analyses (maximum likelihood and Bayesian inference analyses based on LSU, ITS, SSU, *tef*1-α, and *rpb*2 gene combinations) of the new species are provided, along with a comprehensive list of saprobic fungi associated with *Aquilaria* spp.

## ﻿Introduction

*Aquilaria* Lam. (Thymeleaceae) is the main plant genus capable of producing agarwood (Lee and Mohamed 2016; Wang et al. 2018; Li et al. 2021). Currently, there are 21 accepted species in *Aquilaria*, of which 13 have been reported to produce agarwood (Hashim et al. 2016). These trees are tropical and subtropical evergreen broad-leaved trees (Rasool and Mohamed 2016; Xu et al. 2016), widely distributed in Asia, such as Borneo, Cambodia, China, India, Indonesia, Laos, Malaysia, New Guinea, the Philippines, Thailand, and Vietnam, (Wang et al. 2018; 2019). In China, *Aquilariasinensis* is the primary source of agarwood resin (National Pharmacopoeia Committee 2020).

In recent years, a large number of articles have been published on fungi related to *Aquilaria* and agarwood, and it has been found that fungal induction can effectively induce the production of agarwood (Laurence 2013; Liu et al. 2013; Azren et al. 2018; Chen et al. 2018; Subasinghe et al. 2019; Tibpromma et al. 2021; Du et al. 2022a). Du et al. (2022a,b) reported endophytic fungi from agarwood, with the majority belonging to Ascomycota Caval.-Sm. (mainly from *Botryosphaeria* spp., *Fusarium* spp., and *Lasiodiplodia* spp.). In addition, as an important economic plant, the pathogenic fungi of *Aquilaria* have also been extensively studied, and the reported pathogenic fungi mainly belong to Ascomycota, which cause damage to different parts of trees, such as seedling anthracnose (e.g., *Colletotrichumfructicola* Prihast., L. Cai & K.D. Hyde), dieback (e.g., *Lasiodiplodiatheobromae* (Pat.) Griffon & Maubl.), and leaf spot disease (e.g., *Corynesporacassiicola* (Berk. & M.A. Curtis) C.T. Wei) (Borah et al. 2012; Fan et al. 2013; Liao et al. 2018). However, the saprobic fungi of *Aquilaria* have received very little attention. Initially, most saprobic fungi were introduced only based on morphological characteristics, but without molecular data. Du et al. (2022c) initiated exploring and investigating the saprobic fungi of *Aquilaria* spp. based on both morphological and molecular evidence. Some saprobic fungi have recently been discovered on *Aquilaria* spp. in China (Du et al. 2022c, 2023, 2024a, b; Manawasinghe et al. 2024; Tao et al. 2024; Zhang et al. 2024). So far, there are 34 records of saprobic fungi associated with *Aquilaria*, while these saprobes almost all belong to Ascomycota (Du et al. 2024b).

Saprobic fungi, an important component of ecosystems, participate in the carbon cycle by decomposing organic matter (Šnajdr et al. 2011; Li et al. 2022; Niego et al. 2023a). As decomposers, they dominate the litter layer in all forest ecosystems (e.g., tropical, subtropical, temperate, and boreal forests) (Li et al. 2022; Niego et al. 2023b). With increasing attention to ecosystems, extensive research has been conducted on saprobic fungi associated with various plants in recent years. Over 800 taxa have been reported on rubber, of which over half were isolated from leaf and branch litter (Senwanna et al. 2021; Nizamani et al. 2023; Xu et al. 2024). Tian et al. (2024) recently reported 77 saprobic fungi from coconut, pineapple, and rice. In contrast, only 34 records of saprobic fungi are linked to *Aquilaria* spp.; thus, further investigations are required to address this knowledge gap.

In this study, we introduce two new ascomycete species, *Aquilariomycesmaomingensis* (Thyridariaceae, Pleosporales) and *Mangifericomesaquilariae* (Pleosporales genus *incertae sedis*, Wijayawardene et al. 2022; Hyde et al. 2024a) based on morphological studies and multilocus phylogenetic analyses. Comprehensive descriptions, photo plates of macro- and micro-morphological characteristics, and phylogenetic analyses highlighting the placement of new taxa are provided. In addition, a list of *Aquilaria*-associated saprobic fungi is provided.

## ﻿Materials and methods

### ﻿Sampling, examination, and isolation

Specimens were collected from agarwood (*Aquilaria* spp.) plantations in Guangdong and Yunnan provinces, China, and the necessary information was recorded (Rathnayaka et al. 2024). Each specimen was put in plastic bags, recorded collection information, and then transported to the laboratory at Qujing Normal University. Morphological characteristics were examined using an OPTEC SZ650 dissecting stereomicroscope (Chongqing, China), and fungal microstructures were observed and photographed using an OLYMPUS DP74 (Tokyo, Japan) digital camera on an OLYMPUS optical microscope (Tokyo, Japan). Morphological structures were measured in the Tarosoft® Image Framework program v. 1.3, and photo plates were edited in Adobe Photoshop CS3 Extended version 22.0.0 software (Adobe Systems, California, the USA).

Single-spore isolation technique was performed according to the description by Senanayake et al. (2020). The fruiting bodies were cut using sterile blades, and sterile needles were used to pick out the ascospores and place them in ddH_2_O on a glass slide. The ascospores in the water were dispersed into a spore suspension and transferred to potato dextrose agar (PDA) for culture at 23–28 °C for 12–48 hours. Then, the single germinated ascospores were picked up and transferred to a new PDA with recording culture characters.

Specimens were deposited at the Guizhou Medical University (**GMB-W**) and Mycological Herbarium of Zhongkai University of Agriculture and Engineering (**MHZU**), China. Living cultures were deposited in the Guizhou Medical University Culture Collection (**GMBCC**), Guizhou Culture Collection (**GZCC**), and Zhongkai University of Agriculture and Engineering Culture Collection (**ZHKUCC**), China. Facesoffungi (FoF) numbers were registered as described in Jayasiri et al. (2015), and MycoBank numbers (MB) were registered as outlined in MycoBank (http://www.MycoBank.org).

### ﻿DNA extraction, PCR amplification, and sequencing

Molecular studies were carried out according to Tian et al. (2024). Total genomic DNA was extracted from one-month-old fresh fungal mycelium cultured in PDA using a DNA Extraction Kit-BSC14S1 (BioFlux, Hangzhou, P.R. China), following the manufacturer’s instructions. The extracted DNA was stored at 4 °C for the polymerase chain reaction (PCR), while the remaining DNA was maintained at -20 °C for long-term storage. The PCR mixture (25 μL) contains 12.5 μL of 2xMaster Mix (mixture of Easy Taq TM DNA Polymerase, dNTPs, and optimized buffer (Beijing Trans Gen Biotech Co., Chaoyang District, Beijing, China)), 8.5 μL of ddH_2_O, and 1 μL of each forward and reverse primer (10 pM) and 2 μL of DNA template (Tibpromma et al. 2018). The PCR was carried out using the following primers: The internal transcribed spacer (ITS) region was amplified with the primers ITS4 and ITS5 (White et al. 1990); the 28S large subunit (LSU) region was amplified using the primers LR0R and LR5 (Vilgalys and Hester 1990); the 18S small subunit (SSU) region was amplified using the primers NS1 and NS4 (White et al. 1990); the partial translation elongation factor1-alpha (*tef*1-α) gene was amplified using the primers EF1-983F and EF1-2218R (Rehner 2001); and the partial RNA polymerase II subunit (*rpb2*) region was amplified with primers fRPB2-5F and fRPB2-7cR (Liu et al. 1999). The PCR thermal cycle programs for LSU, ITS, SSU, and *tef*1-α were as follows: an initialization step of 94 °C for 3 min, followed by 35 cycles of 94 °C for 30 s, an annealing step at 55 °C for 50 s, an elongation step at 72 °C for 1 min, and a final extension step of 72 °C for 10 min; and the PCR thermal cycle programs for *rpb*2 were as follows: an initialization step of 95 °C for 3 min, followed by 40 cycles of 95 °C for 50 s, an annealing step at 57 °C for 50 s, an elongation step at 72 °C for 90 s, and a final extension step of 72 °C for 10 min. Purification and sequencing of PCR products were carried out using the same primers by Sangon Biotech Co., Kunming, China.

### ﻿Phylogenetic analyses

Phylogenetic analyses were carried out according to Dissanayake et al. (2020). Both reverse and forward sequences generated in this study were assembled using Geneious 9.1.8 (https://www.geneious.com) (Kearse et al. 2012), and newly generated sequences were searched using the BLASTn in NCBI (https://blast.ncbi.nlm.nih.gov/Blast.cgi?PAGE_TYPE=BlastSearch) to identify the most similar taxa. The additional sequences included in the analyses were collected from recent publications (Yang et al. 2022; Du et al. 2024b) and downloaded from GenBank (https://www.ncbi.nlm.nih.gov/WebSub/?form=history&tool=genbank). The FASTA file was created using the OFPT (Zeng et al. 2023) with the protocol used for constructing the Randomized Accelerated Maximum Likelihood (RAxML) and Bayesian Inference analyses (BI). Then FASTA format was converted to PHYLIP (for RAxML) and NEXUS formats (for BI), respectively, using ALTER (http://www.sing-group.org/ALTER/) (Glez-Peña et al. 2010).

The RAxML was generated on the CIPRES Science Gateway (https://www.phylo.org/portal2/login!input.action) (Miller et al. 2010), using RAxML-HPC2 on XSEDE (8.2.12) with 1,000 bootstrap replicates (Stamatakis et al. 2008; Stamatakis 2014) and the GTR+I+G model of evolution and bootstrap supports. The BI tree was performed with MrBayes on XSEDE (3.2.7a) (Ronquist et al. 2012), and the best models of evolution were estimated using MrModeltest v. 2.3 (Nylander et al. 2008). Six simultaneous Markov chains were run for 1,000,000 to 2,000,000 generations, and the tree was sampled every 100^th^ generation. The phylogenetic tree was visualized in FigTree v.1.4.2 (Rambaut 2012) and edited by Microsoft Office PowerPoint 2021 and Adobe Photoshop CS3 Extended version 22.0.0 software (Adobe Systems, California, the USA). All newly generated sequences in this study were deposited to GenBank (https://www.ncbi.nlm.nih.gov/WebSub/?form=history&tool=genbank).

### ﻿A list of saprobic fungi associated with *Aquilaria*

**Figure 1.**
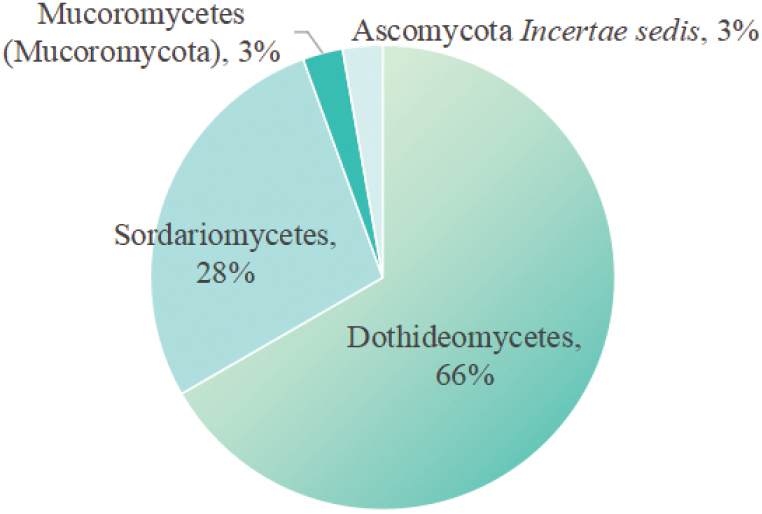
*Aquilaria* spp.-associated saprobic fungi in different classes and *incertae sedis*.

A list of reported saprobic fungi associated with *Aquilaria* spp. is listed in Table 1, and the percentage of different classes is shown in Fig. 1, which includes two new species introduced in this study. This list includes the host and distributions, molecular data, and relevant references of *Aquilaria*-associated saprobic fungi.

**Table 1.** A list of saprobic fungi reported on *Aquilaria* spp.

Host species	Host countries	Phylum	Class	Fungal species	Molecular data	References
*A.agallocha*	Bangladesh	Ascomycota	Sordariomycetes	*Phomopsisaquilariae*	NA	Punithalingam and Gibson 1978
*A.sinensis*	China	Ascomycota	Sordariomycetes	*Allocryptovalsaaquilariae*	ITS and TUB	Chethana et al. 2023
*A.sinensis*	China	Ascomycota	Dothideomycetes	*Aquilariomycesmaomingensis*	ITS, LSU, SSU, *tef*1-α, and *rpb*2	This study
*A.sinensis*	China	Ascomycota	Dothideomycetes	*Barriopsisstevensiana*	ITS, LSU, SSU, and *tef*1-α	Hyde et al. 2024c
*A.sinensis*	China	Ascomycota	Dothideomycetes	*Camarographiumclematidis*	ITS, LSU, SSU, and *tef*1-α	Du et al. 2024b
*A.sinensis*	China	Ascomycota	Dothideomycetes	*Melomastiaaquilariae*	LSU, SSU, and *tef*1-α	Manawasinghe et al. 2024
*A.sinensis*	China	Ascomycota	Dothideomycetes	*Melomastiaguangdongensis*	LSU, SSU, and *tef*1-α	Du et al. 2024a
*A.sinensis*	China	Ascomycota	Dothideomycetes	*Melomastiamaomingensis*	ITS, LSU, SSU, *tef*1-α, and *rpb*2	Du et al. 2024b
*A.sinensis*	China	Ascomycota	Dothideomycetes	*Montagnulaaquilariae*	ITS, LSU, SSU, and *tef*1-α	Hyde et al. 2023
*A.sinensis*	China	Ascomycota	Dothideomycetes	*Nigrogranaaquilariae*	ITS, LSU, SSU, *tef*1-α, and *rpb*2	Du et al. 2024b
*A.sinensis*	China	Ascomycota	Sordariomycetes	*Peroneutypaaquilariae*	ITS and TUB	Du et al. 2022c
*A.sinensis*	China	Ascomycota	Sordariomycetes	*Peroneutypamaomingensis*	ITS, LSU, SSU, *tef*1-α, *rpb*2, and TUB	Du et al. 2024b
*A.sinensis*	China	Ascomycota	Dothideomycetes	*Pseudothyridariellaaquilariae*	ITS, LSU, SSU, *tef*1-α, and *rpb*2	Du et al. 2024b
*A.sinensis*	China	Ascomycota	Dothideomycetes	*Rhytidhysteronthailandicum*	ITS, LSU, SSU, and *tef*1-α	Du et al. 2023
*A.sinensis*	China	Ascomycota	Sordariomycetes	*Triangulariaaquilariae*	ITS, LSU, SSU, *rpb*2, and TUB	Du et al. 2024b
*A.sinensis*	China	Ascomycota	Sordariomycetes	*Allocryptovalsarabenhorstii*	ITS and TUB	Hyde et al. 2024c
*Aquilaria* sp.	China	Ascomycota	Sordariomycetes	*Allocryptovalsacastaneae*	ITS and TUB	Zhang et al. 2024
*Aquilaria* sp.	Thailand	Ascomycota	Dothideomycetes	*Cercosporella* sp.	NA	Subansenee et al. 1985
*Aquilaria* sp.	Thailand	Ascomycota	Sordariomycetes	*Chaetomiumspirale*	NA	Subansenee et al. 1985
*Aquilaria* sp.	Thailand	Ascomycota	Dothideomycetes	*Cladosporium* sp.	NA	Subansenee et al. 1985
*Aquilaria* sp.	China	Ascomycota	Dothideomycetes	*Mangifericomesaquilariae*	ITS, LSU, SSU, *tef*1-α, and *rpb*2	This study
*Aquilaria* sp.	China	Ascomycota	Dothideomycetes	*Melomastiaclematidis*	LSU, SSU, and *tef*1-α	Tao et al. 2024
*Aquilaria* sp.	China	Ascomycota	Dothideomycetes	*Melomastiasinensis*	LSU, SSU, and *tef*1-α	Du et al. 2024a
*Aquilaria* sp.	China	Ascomycota	Dothideomycetes	*Melomastiawinteri*	LSU, SSU, and *tef*1-α	Hyde et al. 2024c
*Aquilaria* sp.	China	Ascomycota	Dothideomycetes	*Melomastiayunnanensis*	LSU, SSU, and *tef*1-α	Du et al. 2024a
*Aquilaria* sp.	China	Ascomycota	Dothideomycetes	*Nigrogranamagnoliae*	ITS, LSU, SSU, and *tef*1-α	Hyde et al. 2024c
*Aquilaria* sp.	Thailand	Ascomycota	Sordariomycetes	*Phialogeniculata* sp.	NA	Subansenee et al. 1985
*Aquilaria* sp.	Thailand	Ascomycota	Dothideomycetes	*Pithomyces* sp.	NA	Subansenee et al. 1985
*Aquilaria* sp.	China	Ascomycota	*Incertae sedis*	*Pseudoacrodictysdeightonii*	ITS, LSU, SSU, and TUB	Hyde et al. 2024c
*Aquilaria* sp.	Thailand	Mucoromycota	Mucoromycetes	*Rhizopus* sp.	NA	Subansenee et al. 1985
*Aquilaria* sp.	China	Ascomycota	Dothideomycetes	*Thyridariaaureobrunnea*	ITS, LSU, *tef*1-α, and *rpb*2	Hyde et al. 2024c
*Aquilaria* sp.	Thailand	Ascomycota	Sordariomycetes	*Trichoderma* sp.	NA	Subansenee et al. 1985
*A.yunnanensis*	China	Ascomycota	Dothideomycetes	*Aquilariomycesaquilariae*	ITS, LSU, SSU, *tef*1-α, and *rpb*2	Du et al. 2024b
*A.yunnanensis*	China	Ascomycota	Dothideomycetes	*Corynesporaaquilariae*	ITS, LSU, SSU, and *tef*1-α	Du et al. 2024b
*A.yunnanensis*	China	Ascomycota	Dothideomycetes	*Parathyridariellaaquilariae*	ITS, LSU, SSU, and *rpb*2	Du et al. 2024b
*A.yunnanensis*	China	Ascomycota	Dothideomycetes	*Phaeoseptumaquilariae*	ITS, LSU, SSU, *tef*1-α, and *rpb*2	Du et al. 2024b

**Notes**: NA = Sequence unavailability.

## ﻿Results

### ﻿Taxonomy

**Thyridariaceae Q. Tian & K.D. Hyde, 2013**

#### ﻿ Aquilariomyces

Taxon classificationFungiPleosporalesThyridariaceae

T.Y. Du, Tibpromma & Karun. 2024

0354648C-CFF0-581C-973E-C0C8FA855A58

##### Notes.

**Figure 2.**
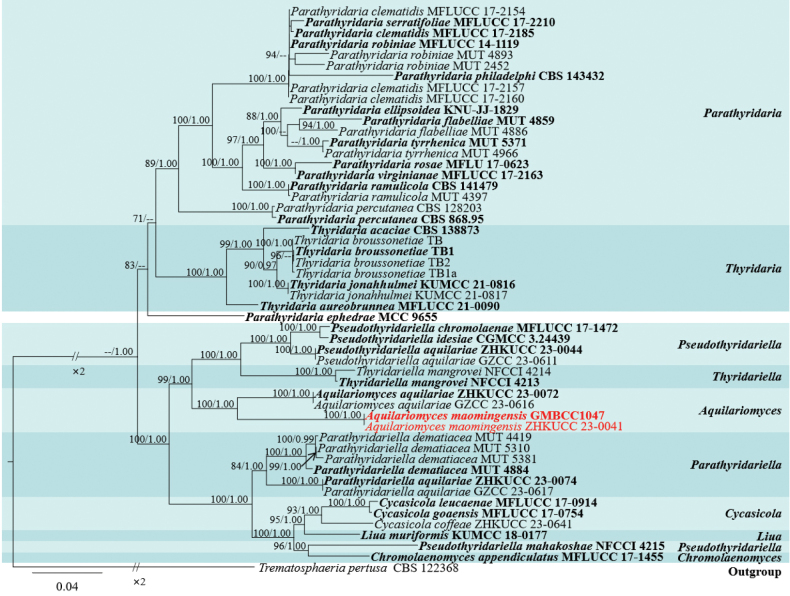
Phylogram generated from maximum likelihood (ML) analysis based on combined LSU, ITS, SSU, *tef*1-α, and *rpb*2 sequence data of 52 taxa, which comprised 4387 base pairs of LSU = 967, ITS = 516, SSU = 872, *rpb*2 = 1024, *tef*1-α = 1008. The best-scoring RAxML tree with a final likelihood value of -31596.745436 is presented. The matrix had 2090 distinct alignment patterns, with 47.13% of undetermined characters or gaps. Estimated base frequencies were as follows: A = 0.249245, C = 0.257277, G = 0.271627, T = 0.221851; substitution rates: AC = 1.210949, AG = 2.948777, AT = 1.318881, CG = 0.897105, CT = 6.444599, GT = 1.0; gamma distribution shape parameter *α* = 0.441696. Bootstrap support values for ML equal to or greater than 70% and clade credibility values equal to or greater than 0.90 from Bayesian inference analysis are labelled at each node. The tree is rooted with *Trematosphaeriapertusa* (CBS 122368). The new isolates are indicated in red, and the ex-type strains are in bold.

*Aquilariomyces* was introduced by Du et al. (2024b) as a monotypic genus to accommodate *Aq.aquilariae* T.Y. Du, Tibpromma & Karun. as the type species, which was collected from *Aquilariayunnanensis* S.C. Huang in Yunnan Province, China. Based on well-separated phylogenetic branches and unique sexual morphs of asci and ascospores, *Aquilariomyces* was introduced as a new genus in Thyridariaceae (Du et al. 2024b). This genus is characterized by globose to subglobose, brown to dark brown, solitary or gregarious ascomata in small groups, immersed under the bark, surrounded by brown to black fluffs; a peridium comprising hyaline to brown cells of ***textura angularis***; hyaline septate, branched, trabeculate pseudoparaphyses, embedded in a gelatinous matrix; 8-spored, bitunicate, clavate, apically rounded asci, with an ocular chamber, and club-shaped, short pedicel; and uniseriate, 1-septate, fusiform to ellipsoidal ascospores, surrounded by mucilaginous sheath, while the asexual morph is not reported. The updated phylogenetic tree of *Aquilariomyces* is shown in Fig. 2.

#### ﻿ Aquilariomyces maomingensis

Taxon classificationFungiPleosporalesThyridariaceae

T.Y. Du, K.D. Hyde, Tibpromma & Karun. sp. nov.

9AB1A9EB-3507-5922-B58C-74699725C4C0

856409

Facesoffungi Number: FoF16960

Fig. 3

##### Etymology.

Named after the location “Maoming,” where the holotype was collected.

##### Holotype.

MHZU 23-0022.

##### Description.

***Saprobic*** on decaying branch of *Aquilariasinensis*. **Sexual morph: *Ascomata*** (excluding necks) 250–450 μm high × 200–500 μm diam. (x̄ = 366 × 350 μm, n = 5), solitary or gregarious in small groups, brown to dark brown, surrounded by short brown to black fluffs, immersed, slightly raised under the bark, globose to subglobose, sometimes ovoid, ostiolate. ***Ostiolar canal*** 250–280 µm long × 150–200 µm wide (x̄ = 263 × 180 µm, n = 10), cylindrical to elliptical, usually straight, dark brown to black necks with periphyses. ***Peridium*** 15–70 μm (x̄ = 31 μm, n = 30) wide, comprising 3–5 layers of pale brown to brown cells of ***textura angularis*** to ***textura prismatica***, fusing with the host tissue. ***Hamathecium*** comprising 1 μm wide, hyaline, septate, branched, numerous, trabeculate pseudoparaphyses (*sensu*Liew et al. 2000), embedded in a gelatinous matrix. ***Asci*** 100–140 × 21–25 μm (x̄ = 123 × 23 μm, n = 30), bitunicate, 8-spored, thick-walled, clavate, apically rounded, with an ocular chamber, short pedicel, some club-shaped. ***Ascospores*** 20–36 × 9–15 μm (x̄ = 30 × 13 μm, n = 30), uniseriate, 1-septate, fusiform to ellipsoidal, conical at both ends or round, constricted at the septum, upper cells are slightly larger than below cells, rough-walled, with several guttules and granules, hyaline to pale yellow when immature and surrounded by a mucilaginous sheath, later become yellow-brown and without a mucilaginous sheath. **Asexual morph**: Undetermined.

**Figure 3.**
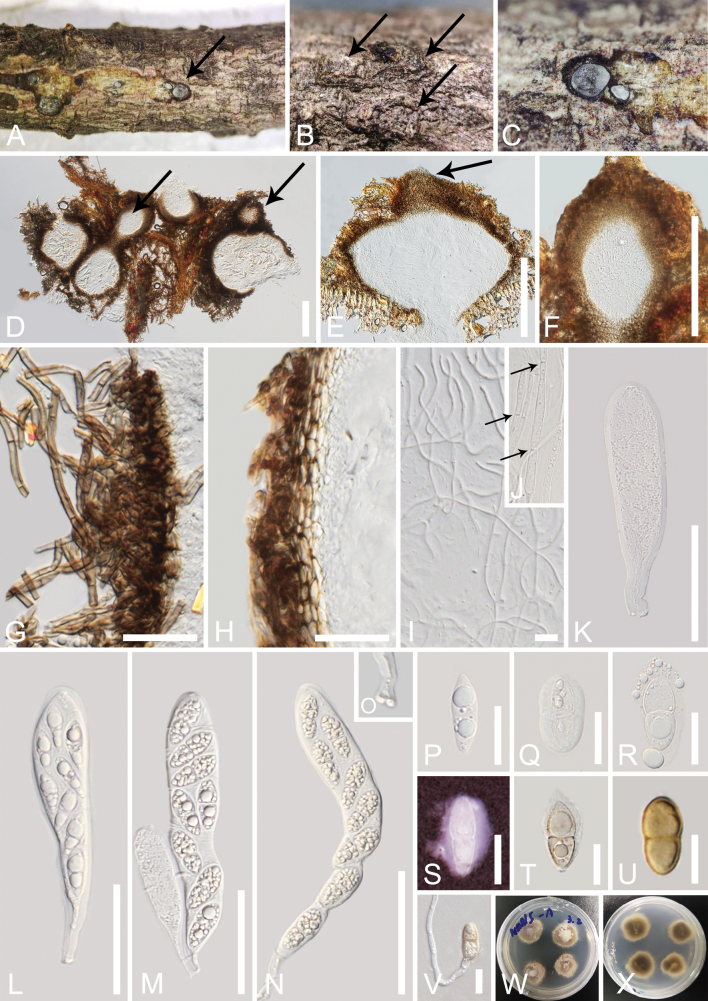
*Aquilariomycesmaomingensis* (MHZU 23-0022, holotype) **A–C** appearance of ascomata on the host (the arrows indicate ascomata) **D, E** vertical sections through the ascomata (the arrows indicate ostioles) **F** ostiole with periphyses **G** short fluffs around the periphery of the ascomata **H** peridium **I, J** trabeculae pseudoparaphyses (**J** the arrows indicate septate pseudoparaphyses) **K–N** asci **O** a club-shaped pedicel **P–U** ascospores (**S** stained with Indian ink) **V** germinated ascospore **W, X** colonies on PDA obverse and reverse views. Scale bars: 200 µm (**D–F**); 30 µm (**G, H**); 10 µm (**I**); 50 µm (**K–N**); 20 µm (**P–V**).

##### Culture characteristics.

Ascospores germinated on PDA after 12 hours, and germ tubes were produced from one or both ends. ***Colonies*** on PDA reaching 2–3 cm diam. after two weeks at 23–28 °C. Colonies obverse: dense, circular, or irregular, cream to brown, umbonate, raised at the center, filamentous edge. Colonies reverse dark brown to black at the center and cream to light brown at the margin.

##### Material examined.

China • Guangdong Province, Maoming City, Dianbai District, Poxin Town, 21°34'25"N, 111°7'43"E, on a dead branch of *Aquilariasinensis* (Thymelaeaceae), 3 June 2022, T.Y. Du, MMA15 (MHZU 23-0022, holotype), ex-type living culture, GMBCC1047; additional living culture, ZHKUCC 23-0041.

##### GenBank numbers.

GMBCC1047: ITS = PQ604643, LSU = PQ604620, SSU = PQ604624, *tef*1-α = PQ612415, *rpb*2 = PQ612419; ZHKUCC 23-0041: ITS = PQ604644, LSU = PQ604621, SSU = PQ604625, *tef*1-α = PQ612416, *rpb*2 = PQ612420.

##### Notes.

**Figure 4.**
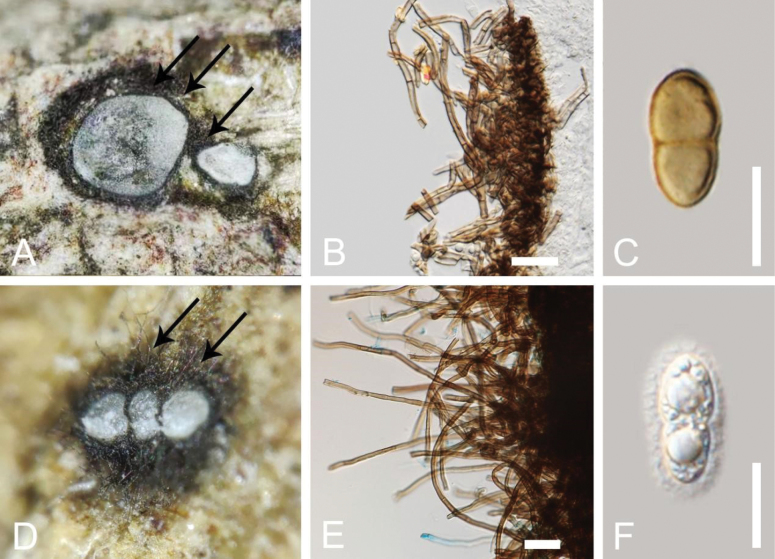
Comparison of morphological structure between *Aquilariomycesmaomingensis* and *Aq.aquilariae*. *Aquilariomycesmaomingensis* (MHZU 23-0022 holotype) **A** ascomata wrapped in short fluffs **B** micrograph of fluffs **C** brown ascospores. *Aquilariomycesaquilariae* (MHZU 23-0036, holotype) (Du et al. 2024b) **D** ascomata wrapped in long fluffs **E** micrograph of fluffs **F** hyaline ascospores. Scale bars: 20 µm (**B, C, E, F**).

In the present phylogenetic analyses, our new collection, *Aquilariomycesmaomingensis*, formed a well-separated sister lineage to *Aq.aquilariae* (ZHKUCC 23-0072 and GZCC 23-0616) with 100% ML and 1.00 BYPP statistical support (Fig. 2). *Aquilariomycesmaomingensis* shares similar morphological characteristics with *Aq.aquilariae* (MHZU 23-0036, holotype) in having immersed, globose to subglobose ascomata, numerous, septate, branched, trabeculate pseudoparaphyses in a gelatinous matrix, clavate asci, with short and club-shaped pedicel, and uniseriate fusiform to ellipsoidal, 1-septate, ascospores, constricted at the septum, and surrounded by a mucilaginous sheath (Du et al. 2024b). However, *Aq.maomingensis* (MHZU 23-0022) differs from *Aq.aquilariae* (MHZU 23-0036) in its ascomata and ascospore characters. *Aquilariomycesmaomingensis* has ascomata surrounded by short fluffs, slightly raised under the bark, and brown mature ascospores, while *Aq.aquilariae* (MHZU 23-0036) has inconspicuous ascomata, surrounded by long fluffs, and hyaline mature ascospores (Du et al. 2024b). A comparison of the main morphological structures between *Aquilariomycesmaomingensis* and *Aq.Aquilariae* is shown in Fig. 4.

According to the phylogenetic analysis of the present study, both *Aquilariomyces* species clustered in Thyridariaceae, a family characterized by trabeculate or cellular pseudoparaphyses. Trabeculate pseudoparaphyses are characterized by narrow, thread-like, apparently nonseptate, branched, and anastomosing or unbranched above the asci and embedded in a gelatinous matrix (Liew et al. 2000; Hongsanan et al. 2020). This type of pseudoparaphyses is found in *Aquilariomyces* (Fig. 3I). Trabeculae were considered important at the Dothideomycetes O.E. Erikss. & Winka in earlier classifications; thus, Melanommatales was defined as having trabeculae (Barr 1983). However, Liew et al. (2000) showed that trabeculae were not important at the order level and probably were important at the family level (or even species). Thyridariaceae comprises nine genera: *Aquilariomyces*, *Chromolaenomyces* Mapook & K.D. Hyde, *Cycasicola* Wanas., E.B.G. Jones & K.D. Hyde, *Liua* Phookamsak & K.D. Hyde, *Parathyridaria* Jaklitsch & Voglmayr, *Parathyridariella* Prigione, A. Poli, E. Bovio & Varese, *Pseudothyridariella* Mapook & K.D. Hyde, *Thyridaria* Sacc., and *Thyridariella* Devadatha, V.V. Sarma, K.D. Hyde, Wanas. & E.B.G. Jones (Wijayawardene et al. 2022; Du et al. 2024b). Among these genera, *Aquilariomyces* (Du et al. 2024b), *Parathyridaria* (Jaklitsch and Voglmayr 2016), and *Thyridaria* (Jaklitsch and Voglmayr 2016) have trabeculate pseudoparaphyses; *Chromolaenomyces* (Mapook et al. 2020), *Pseudothyridariella* (Mapook et al. 2020), and *Thyridariella* (Devadatha et al. 2018) have cellular pseudoparaphyses, while pseudoparaphyses of this type have not been reported yet in other genera, *viz. Cycasicola*, *Liua*, and *Parathyridariella*. We believe pseudoparaphyses type is one of the important characters at the genus level.

The base pair differences in the LSU, ITS, SSU, *tef*1-α, and *rpb*2 genes (without gaps) between our new collection and *Aq.aquilariae* (ZHKUCC 23-0072, ex-type) were also compared. The results showed that there are 3.1% nucleotide differences (28/912 bp) in LSU; in comparison, ITS has 12.3% nucleotide differences (67/544 bp), SSU has 0.3% nucleotide differences (3/873 bp), *tef*1-α has 7.5% nucleotide differences (76/1008 bp), and *rpb*2 has 10.6% nucleotide differences (109/1025 bp). These comparisons indicate minor differences in SSU and LSU, while there are considerable base differences in ITS, *tef*1-α, and *rpb*2. Therefore, we introduce our new collection as a new species, *Aq.maomingensis*, based on a polyphasic approach, according to the guidelines of Maharachchikumbura et al. (2021). *Aquilariomycesmaomingensis*, the second *Aquilariomyces* species, was collected from the same host genus and country (*Aquilariasinensis*, China) as the first.

### ﻿Pleosporales genera *incertae sedis*

#### ﻿ Mangifericomes

Taxon classificationFungiPleosporalesThyridariaceae

E.F. Yang & Tibpromma, 2022

FB92B032-8512-51EE-BC26-3F4A6C83A5B5

##### Notes.

**Figure 5.**
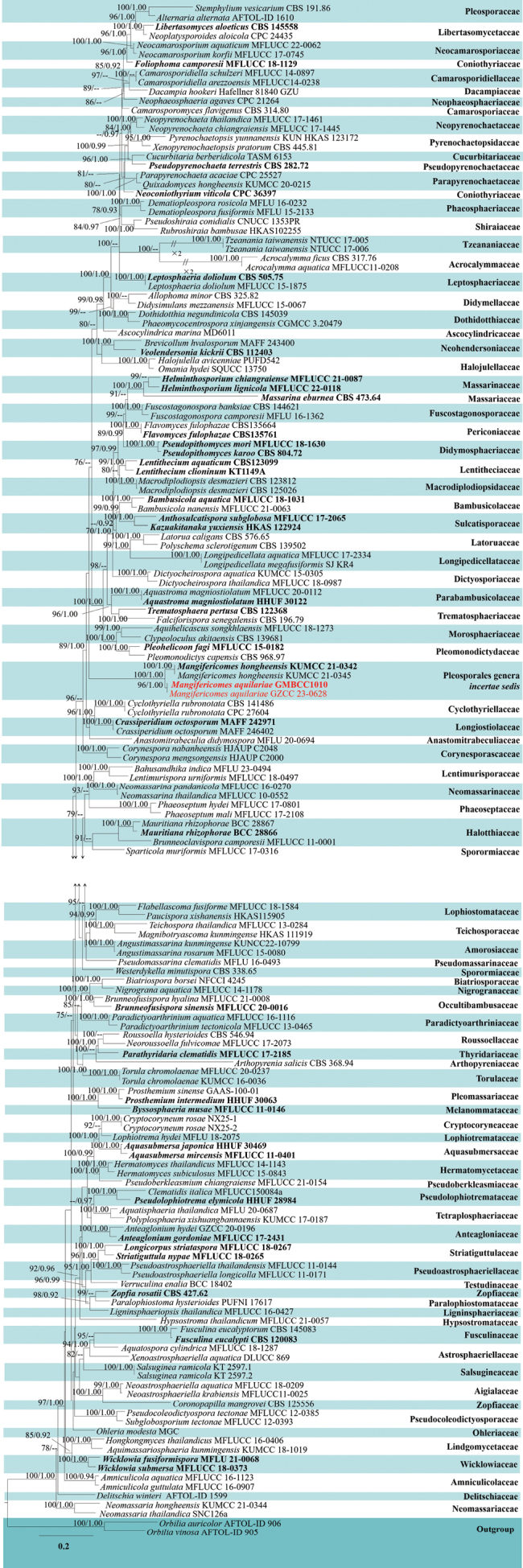
Phylogram generated from ML analysis of Pleosporales based on combined LSU, ITS, SSU, *tef*1-α, and *rpb*2 sequence data of 159 taxa, which comprised 4584 base pairs of LSU = 1201, ITS = 534, SSU = 1018, *rpb*2 = 930, *tef*1-α = 901. The best-scoring RAxML tree with a final likelihood value of -91408.743991 is presented. The matrix had 3121 distinct alignment patterns, with 41.32% of undetermined characters or gaps. Estimated base frequencies were as follows: A = 0.246701, C = 0.244302, G = 0.274425, T = 0.234571; substitution rates: AC = 1.391138, AG = 3.919388, AT = 1.582417, CG = 1.094328, CT = 7.887132, GT = 1.0; gamma distribution shape parameter *α* = 0.418663. Bootstrap support values for maximum likelihood (ML) equal to or greater than 70% and clade credibility values greater than 0.90 from Bayesian inference analysis are labelled at each node. The tree is rooted with *Orbiliaauricolor* (AFTOL-ID 906) and *O.vinosa* (AFTOL-ID 905). The new isolates are indicated in red, and the ex-type strains are in bold.

*Mangifericomes* was established by Yang et al. (2022) as a monotypic genus in the Pleosporales genera *incertae sedis* to accommodate *M.hongheensis* E.F. Yang and Tibpromma as type species, which was isolated from *Mangiferaindica* L. in China. *Mangifericomes* is characterized by immersed or semi-immersed, globose to subglobose, dark brown to black ascomata with or without ostioles; a hamathecium comprising filiform, hyaline, septate, branched cellular pseudoparaphyses (*sensu*Liew et al. 2000); 8-spored, bitunicate, cylindrical-clavate, pedicellate asci; and ellipsoid, muriform, pale brown to brown ascospores, wrapped in a gelatinous sheath (Yang et al. 2022). The updated phylogenetic tree of *Mangifericomes* is shown in Fig. 5.

#### ﻿ Mangifericomes aquilariae

Taxon classificationFungiPleosporalesThyridariaceae

T.Y. Du, K.D. Hyde, Tibpromma & Karun. sp. nov.

F92CA690-8D21-50B1-B93E-E087EFFB5B9A

856410

Facesoffungi Number: FoF16961

Fig. 6

##### Etymology.

Named after the host genus “*Aquilaria*,” from which the holotype was collected.

##### Holotype.

GMB-W 1008.

##### Description.

***Saprobic*** on decaying branch of *Aquilaria* sp. **Sexual morph: *Ascomata*** 280–460 μm high × 250–510 μm diam. (x̄ = 375 × 380 μm, n = 10), globose to subglobose, brown to dark brown, gregarious, immersed, inconspicuous on host surface, ostiolate. ***Peridium*** 20–70 μm (x̄ = 40 μm, n = 20) wide, comprising 5–7 layers of hyaline to pale brown cells of ***textura angularis*** to ***textura prismatica***, fusing with the host tissue. ***Hamathecium*** 2.5 μm wide, hyaline, fascicular, septate, branched, numerous, cellular pseudoparaphyses, embedded in a glutinous matrix. ***Asci*** 170–265 × 32–50 μm (x̄ = 216 × 40 μm, n = 30), bitunicate, fissitunicate, 8-spored, cylindric-clavate, with short pedicel, apically rounded, with an ocular chamber. ***Asco­spores*** 40–53 × 18–23 μm (x̄ = 47 × 20 μm, n = 30), muriform, uniseriate, hyaline and later become golden yellow, pale brown to dark brown, ellipsoid, slightly curved to straight, rough-walled, slightly wider near apex, apically rounded, 10–13-transversally septate, and 3–6-longitudinal septa, slightly constricted at the septum, surrounded by a 6.5–15 µm wide gelatinous sheath. **Asexual morph**: Undetermined.

**Figure 6.**
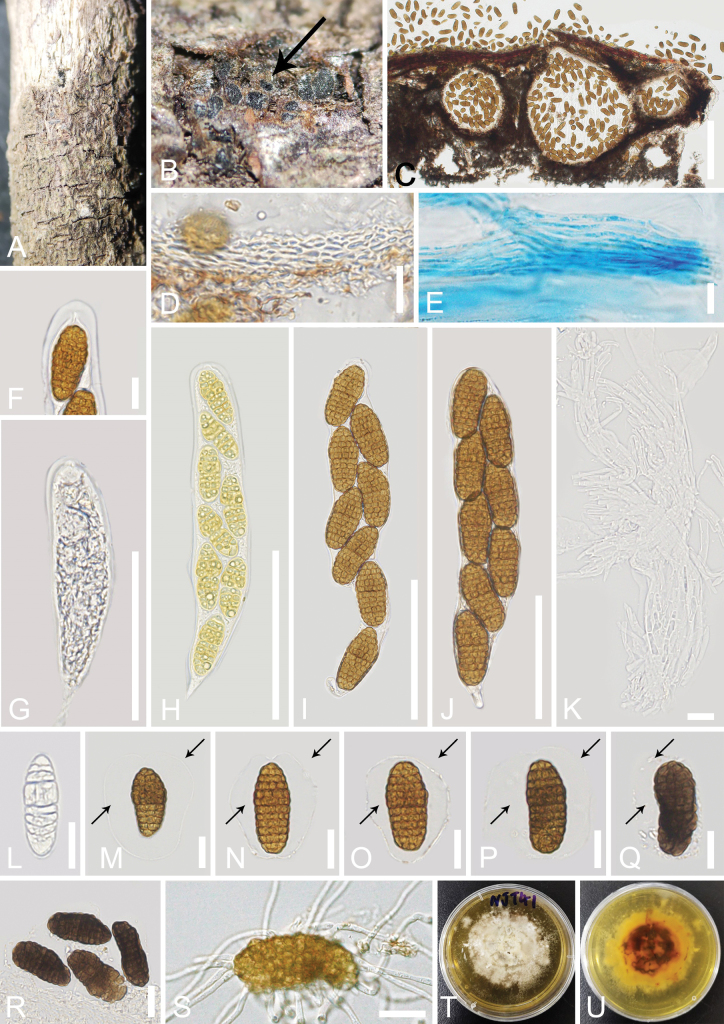
*Mangifericomesaquilariae* (GMB-W 1008, holotype) **A, B** ascomata on the host (the arrow indicates the cross-section of the ascomata) **C** vertical sections through the ascomata **D** peridium **E, K** cellular pseudoparaphyses (**E** stained with cotton blue) **F** ocular chamber of asci **G–J** asci **L–R** ascospores (the arrows indicate the sheath of the ascospores) **S** germinated ascospore **T, U** colony on PDA obverse and reverse views. Scale bars: 200 µm (**C**); 20 µm (**D, G, L–S**); 10 µm (**E, K**); 100 µm (**F, H–J**).

##### Culture characteristics.

Ascospores germinated on PDA after 24 hours, and germ tubes were produced from each cell. ***Colonies*** on PDA reaching 5 cm diam., after four weeks at 23–28 °C. Colonies obverse: loose, circular or irregular, white-cream, slightly raised at the center, filamentous edge. Colonies reverse reddish-brown at the center and cream to light yellow towards the periphery.

##### Material examined.

China • Yunnan Province, Nujiang Prefecture, Lushui City, Liuku Town, 25°48'30"N, 98°51'5"E, on a dead branch of *Aquilaria* sp. (Thymelaeaceae), 21 April 2023, T.Y. Du, NJT41 (GMB-W 1008, holotype), ex-type living culture, GMBCC1010; additional living culture, GZCC 23-0628.

##### GenBank numbers.

GMBCC1010: ITS = PQ604645, LSU = PQ604622, *tef*1-α = PQ612417, *rpb*2 = PQ612421; GZCC 23-0628: ITS = PQ604646, LSU = PQ604623, *tef*1-α = PQ612418, *rpb*2 = PQ612422.

##### Notes.

In the present phylogenetic analyses, our new collection, *Mangifericomesaquilariae*, formed a well-separated sister lineage to *M.hongheensis* (KUMCC 21-0342 and KUMUCC 21-0345) with 100% ML and 1.00 BYPP statistical support (Fig. 5). *Mangifericomesaquilariae* shares similar morphological characteristics with *M.hongheensis* (HKAS 1221888, holotype) in having globose to subglobose, brown to dark brown, ostiolate ascomata, bitunicate asci with fissitunicate, 8-spored, cylindrical-clavate, and muriform ascospores, ellipsoid, pale brown to dark brown, slightly wider near the apex, surrounded by a gelatinous sheath (Yang et al. 2022). However, *M.aquilariae* (GMB-W 1008) differs from *M.hongheensis* (HKAS 1221888) by its immersed ascomata, peridium comprising ***textura prismatica*** to ***textura angularis*** cells, fascicular, and numerous pseudoparaphyses, and ascospores that are 10–13-transversally septate, 3–6-longitudinal septa, while ascomata of *M.hongheensis* (HKAS 1221888) are semi-immersed to fully immersed, peridium comprising ***textura angularis*** to ***textura globosa*** cells, sparse pseudoparaphyses, and ascospores that are 7–11-transversally septate, 5–8-longitudinal septa (Yang et al. 2022). In addition, the base pair differences of the LSU, ITS, *tef*1-α, and *rpb*2 genes (without gaps) between our new collection and *M.hongheensis* (KUMCC 21-0342, ex-type) were compared, while the SSU of our new collection is not available. The results showed that there are 0.8% nucleotide differences (7/864 bp) in LSU, while ITS has 3.4% nucleotide differences (18/533 bp), *tef*1-α has 3.2% nucleotide differences (31/962 bp), and *rpb*2 has 4.5% nucleotide differences (41/905 bp). These comparisons indicate that they display minor differences on LSU while displaying significant base differences on ITS, *tef*1-α, and *rpb*2. Therefore, we introduce our new collection (from *Aquilaria* sp. in China) as a new species, *M.aquilariae*, based on a polyphasic approach according to the guidelines of Maharachchikumbura et al. (2021). In addition, this study introduces the second *Mangifericomes* species in the genus and the first *Mangifericomes* species collected from *Aquilaria*.

## ﻿Discussion

*Aquilaria*, the primary genus that produces agarwood, is a plant of significant medicinal and economic value, with 13 species known to produce agarwood (Hashim et al. 2016). As Table 1 and Fig. 1 show, saprobic fungi associated with *Aquilaria* mainly belong to Dothideomycetes and Sordariomycetes O.E. Erikss. & Winka of Ascomycota, with only one taxon reported in Mucoromycetes Doweld of Mucoromycota Doweld. This is also consistent in both endophytic and pathogenic fungi of *Aquilaria* spp. (Du et al. 2022a,b). The currently recorded distribution range of these saprobic fungi is mainly in China (28 records, including this study), Thailand (seven records), and only one record in Bangladesh. This is mainly because *Aquilaria* is a tropical tree genus, mainly distributed in tropical regions. Among these records, eight species (*Cercosporella* sp., *Chaetomiumspirale*, *Cladosporium* sp., *Phialogeniculata* sp., *Phomopsisaquilariae*, *Pithomyces* sp., *Rhizopus* sp., and *Trichoderma* sp.) lack molecular data and detailed morphological descriptions. It is necessary to explore saprobic fungi associated with *Aquilaria* spp. with more collections in broader geographical regions.

A thorough investigation and systematic sampling in this study resulted in the authentication of two new species of saprobic fungi on *Aquilaria* from China: *Aquilariomycesmaomingensis* on *Aquilariasinensis* from Maoming City, Guangdong Province in June 2022, and *Mangifericomesaquilariae* on *Aquilaria* sp. from Nujiang, Yunnan Province in April 2023. In the genus *Aquilariomyces*, *Aq.aquilariae* was collected from *Aquilariayunnanensis* in Yunnan Province (Du et al. 2024b). In this study, *Aq.maomingensis* is introduced as the second species in *Aquilariomyces*, which was collected from *Aquilariasinensis* in Guangdong Province. These two species come from *Aquilaria* trees in tropical regions. This data may indicate that this genus exhibits host-specificity. However, only two species have been discovered in this genus, which is insufficient to confirm this feature, and this needs more research.

This study provides a phylogenetic tree (Fig. 5) of Pleosporales to determine the position of *Mangifericomes*. After multiple constructions of multi-gene phylogenetic trees, we found that the placement of *Mangifericomes* is highly unstable and has low statistical support with other branches. Therefore, we still classify it as a Pleosporales genus *incertae sedis* (Wijayawardene et al. 2022), and more samples of the genus need to be collected to determine the taxonomic placement. *Mangifericomesaquilariae* is morphologically consistent with the monotypic genus introduced by Yang et al. (2022) and also forms a distinguishable sister branch with the type species *M.hongheensis* in the phylogenetic tree (Fig. 5). *Mangifericomesaquilariae* differs from *M.hongheensis* in morphology and significant differences in the comparison of basepair fragments (Jeewon and Hyde 2016). *Mangifericomeshongheensis* was found on *Mangiferaindica* in Honghe, Yunnan Province, while *M.aquilariae* was found on *Aquilaria* sp. in Nujiang, Yunnan Province. Currently, both species have been isolated from broad-leaved tree species in tropical regions of China. Further research is needed to explore whether the distribution range of this genus is related to tropical plants.

Most members of Ascomycota are saprobes; the phylum is one of the most representative fungal communities involved in saprobic behavior (Benny et al. 2001; Li et al. 2022). The abundance of Ascomycota is higher in tropical forests than in other forest ecosystems (Tedersoo et al. 2014; Li et al. 2022; Hyde et al. 2024b). In order to more systematically fill the gap where only a few saprophytic fungi have been found in *Aquilaria*, in this study, we introduce two new terrestrial saprobic fungal species belonging to the Pleosporales in Ascomycota, which enrich the diversity of saprobic fungi on *Aquilaria*. For the first time, this study lists a comprehensive set of saprobic fungi associated with *Aquilaria* spp., laying a solid foundation for future research on taxa associated with *Aquilaria*.

## Supplementary Material

XML Treatment for Aquilariomyces

XML Treatment for Aquilariomyces maomingensis

XML Treatment for Mangifericomes

XML Treatment for Mangifericomes aquilariae

## ﻿Additional information

### Author ORCIDs

Tian-Ye Du https://orcid.org/0000-0003-2105-1803

Samantha C. Karunarathna https://orcid.org/0000-0001-7080-0781

Kevin D. Hyde https://orcid.org/0000-0002-2191-0762

Somrudee Nilthong https://orcid.org/0000-0002-7454-5826

Ausana Mapook https://orcid.org/0000-0001-7929-2429

Dong-Qin Dai https://orcid.org/0000-0001-8935-8807

Kunhiraman C. Rajeshkumar https://orcid.org/0000-0003-0401-8294

Abdallah M. Elgorban https://orcid.org/0000-0003-3664-7853

Li-Su Han https://orcid.org/0000-0001-5380-9928

Hao-Han Wang https://orcid.org/0000-0002-2128-7894

Saowaluck Tibpromma https://orcid.org/0000-0002-4706-6547

### Data availability

All of the data that support the findings of this study are available in the main text.

## References

[B1] Azren PD Lee SY Emang D Mohamed R (2018) History and perspectives of induction technology for agarwood production from cultivated *Aquilaria* in Asia: A review. Journal of Forestry Research 30(1): 1–11. 10.1007/s11676-018-0627-4

[B2] Barr ME (1983) Muriform ascospores in class ascomycetes. Mycotaxon 18: 149–157.

[B3] Benny GL Humber RA Morton JB (2001) Zygomycota: Zygomycetes. Systematics and Evolution (Vol. 7a). Springer, Berlin/Heidelberg, 113–146. 10.1007/978-3-662-10376-0_6

[B4] Borah RK Ahmed FS Sarmah GS Gogoi B (2012) A new record of leaf spot disease on *Aquilariamalaccensis* Lamk. India. Asian Journal of Plant Pathology 6(2): 48–51. 10.3923/ajppaj.2012.48.51

[B5] Chen XY Liu YY Yang Y Feng J Liu PW Sui C Wei JH (2018) Trunk surface agarwood-inducing technique with *Rigidoporusvinctus*: An efficient novel method for agarwood production. PLoS ONE 13(6): e0198111. 10.1371/journal.pone.0198111 PMC5983524 29856792

[B6] Chethana KWT Rathnayaka AR Samarakoon BC Wu N Wijesinghe SN Yasanthika WAE Sysouphanthong P Thiyagaraja V Armand A Lestari AS Madagammana AD Ediriweera AN Prematunga C Li CJ-Y Tennakoon DS Gomdola D Marasinghe DS Bundhun D Pem D Ren G Zhao H Su HL Win H Li H Lu L Calabon MS Samarakoon MC Chaiwan N Huanraluek N Samaradiwakara NP Kularathnage ND Abeywickrama PD Perera RH Tang SM Du T-Y Punyaboon W Ma X Yang YH Tun ZL Bhunjun CS Manawasinghe IS Senanayake IC Liu J-K Tibpromma S Wadduwage KS Wijayalath WHDN Raspé O Bulgakov TS Camporesi E Promputtha I Hyde KD (2023) AJOM new records and collections of fungi: 151–200. Asian Journal of Mycology 6: 89–243. 10.5943/ajom/3/1/3

[B7] Devadatha B Sarma VV Jeewon R Wanasinghe DN Hyde KD Jones EBG (2018) *Thyridariella*, a novel marine fungal genus from India: Morphological characterization and phylogeny inferred from multigene DNA sequence analyses. Mycological Progress 17(7): 791–804. 10.1007/s11557-018-1387-4

[B8] Dissanayake AJ Bhunjun CS Maharachchikumbura SSN Liu JK (2020) Applied aspects of methods to infer phylogenetic relationships amongst fungi. Mycosphere 11(1): 2652–2676. 10.5943/mycosphere/11/1/18

[B9] Du TY Dao CJ Mapook A Stephenson SL Elgorban AM Al‐Rejaie S Suwannarach N Karunarathna SC Tibpromma S (2022a) Diversity and biosynthetic activities of agarwood associated fungi. Diversity 14(3): 211. 10.3390/d14030211

[B10] Du TY Karunarathna SC Hyde KD Mapook A Wariss HM Aluthwattha ST Wang YH Mortimer PE Xu JC Tibpromma S (2022b) The endophytic fungi of *Aquilariasinensis* from southern China. Fungal Biotec 2: 1–15.

[B11] Du TY Karunarathna SC Zhang X Dai DQ Gao Y Mapook A Tibpromma S (2022c) Morphology and multigene phylogeny revealed *Peroneutypaaquilariae* sp. nov. (Diatrypaceae, Xylariales) from *Aquilariasinensis* in Yunnan Province. China Studies in Fungi 7(1): 18. 10.48130/SIF-2022-0018

[B12] Du TY Dai DQ Mapook A Lu L Stephenson SL Suwannarach N Elgorban AM Al-Rejaie S Karunarathna SC Tibpromma S (2023) Additions to *Rhytidhysteron* (Hysteriales, Dothideomycetes) in China. Journal of Fungi (Basel, Switzerland) 9(2): 148. 10.3390/jof9020148 36836263 PMC9958654

[B13] Du TY Karunarathna SC Tibpromma S Hyde KD Nilthong S Mapook A Liu XF Dai DQ Niu C Elgorban AM Chukeatirote E Wang HH (2024a) *Melomastia* (Dothideomycetes, Ascomycota) species associated with Chinese *Aquilaria* spp. MycoKeys 111: 65–86. 10.3897/mycokeys.111.137898 39669742 PMC11635358

[B14] Du TY Tibpromma S Hyde KD Mapook A Dai DQ Zhang GQ Stephenson SL Suwannarach N Kumla J Elgorban AM Rajeshkumar KC Maharachchikumbura SSN Li Q Karunarathna SC (2024b) The polyphasic approach reveals 10 novel and one known Ascomycota taxa from terrestrial agarwood‐producing trees. Journal of Systematics and Evolution, 1–38. 10.1111/jse.13037

[B15] Fan MC Yeh HC Hong CF (2013) First report of *Lasiodiplodiatheobromae* causing dieback of *Aquilariasinensis* in Taiwan. Plant Disease 97(5): 690–690. 10.1094/PDIS-10-12-0998-PDN 30722198

[B16] Glez‐Peña D Gómez‐Blanco D Reboiro‐Jato M Fdez‐Riverola F Posada D (2010) ALTER, program‐oriented conversion of DNA and protein alignments. Nucleic Acids Research 38: W14–18. 10.1093/nar/gkq321 PMC2896128 20439312

[B17] Hashim YZHY Kerr PG Abbas P Salleh HM (2016) *Aquilaria* spp. (agarwood) as source of health beneficial compounds: A review of traditional use, phytochemistry and pharmacology. Journal of Ethnopharmacology 189: 331–360. 10.1016/j.jep.2016.06.055 27343768

[B18] Hongsanan S Hyde KD Phookamsak R Wanasinghe DN McKenzie EHC Sarma VV Boonmee S Lücking R Pem D Bhat JD Liu N Tennakoon DS Karunarathna A Jiang SH Jones EBG Phillips AJL Manawasinghe I Tibpromma S Jayasiri SC Sandamali D Jayawardena RS Wijayawardene NN Ekanayaka AH Jeewon R Lu YZ Dissanayake AJ Zeng XY Luo ZL Tian Q Phukhamsakda C Thambugala KM Dai DQ Chethana TKW Ertz D Doilom M Liu JK Pérez-Ortega S Suija A Senwanna C Wijesinghe SN Konta S Niranjan M Zhang SN Ariyawansa HA Jiang HB Zhang JF de Silva NI Thiyagaraja V Zhang H Bezerra JDP Miranda-Gonzáles R Aptroot A Kashiwadani H Harishchandra D Abeywickrama PD Bao DF Devadatha B Wu HX Moon KH Gueidan C Schumm F Bundhun D Mapook A Monkai J Chomnunti P Samarakoon MC Suetrong S Chaiwan N Dayarathne MC Jing Y Rathnayaka AR Bhunjun CS Xu JC Zheng JS Liu G Feng Y Xie N (2020) Refined families of Dothideomycetes: Orders and families incertae sedis in Dothideomycetes. Fungal Diversity 105(1): 17–318. 10.1007/s13225-020-00462-6

[B19] Hyde KD Norphanphoun C Ma J Yang HD Zhang JY Du TY Gao Y Gomes de Farias AR He SC He YK Li CJY Li JY Liu XF Lu L Su HL Tang X Tian XG Wang SY Wei DP Xu RF Xu RJ Yang YY Zhang F Zhang Q Bahkali AH Boonmee S Chethana KWT Jayawardena RS Lu YZ Karunarathna SC Tibpromma S Wang Y Zhao Q (2023) Mycosphere notes 387–412 – novel species of fungal taxa from around the world. Mycosphere 14(1): 663–744. 10.5943/mycosphere/14/1/8

[B20] Hyde KD Baldrian P Chen Y Chethana KWT de Hoog S Doilom M de Farias AR Goncalves MF Gonkhom D Gui H Hilário S (2024a) Current trends, limitations and future research in the fungi. Fungal Diversity 125(1): 1–71. 10.1007/s13225-023-00532-5

[B21] Hyde KD Noorabadi MT Thiyagaraja V He MQ Johnston PR Wijesinghe SN Armand A Biketova AY Chethana KWT Erdoğdu M Ge ZW Groenewald JZ Hongsanan S Kušan I Leontyev DV Li DW Lin CG Liu NG Maharachchikumbura SSN Matočec N May TW McKenzie EHC Mešić A Perera RH Phukhamsakda C Piątek M Samarakoon MC Selcuk F Senanayake IC Tanney JB Tian Q Vizzini A Wanasinghe DN Wannasawang N Wijayawardene NN Zhao RL Abdel-Wahab MA Abdollahzadeh J (2024b) The 2024 Outline of Fungi and fungus-like taxa. Mycosphere 15(1): 5146–6239. 10.5943/mycosphere/15/1/25

[B22] Hyde KD Wijesinghe SN Afshari N Aumentado HD Bhunjun CS Boonmee S Camporesi E Chethana KWT Doilom M Du TY Farias ARG Gao Y Jayawardena RS Karimi O Karunarathna SC Kularathnage ND Lestari AS Li CJY Li YX Liao CF Liu XF Lu L Lu YZ Luo ZL Ma J Mamarabadi M Manawasinghe IS Mapook A Mi LX Niranjan M Senanayake IC Shen HW Su HL Tibpromma S Xu RJ Yan JY Yang YH Yang YY Yu FQ Kang JC Zhang JY (2024c) Mycosphere Notes 469–520. Mycosphere 15(1): 1294–1454. 10.5943/mycosphere/15/1/11

[B23] Jaklitsch WM Voglmayr H (2016) Hidden diversity in *Thyridaria* and a new circumscription of the Thyridariaceae. Studies in Mycology 85(1): 35–64. 10.1016/j.simyco.2016.09.002 27872508 PMC5109270

[B24] Jayasiri SC Hyde KD Ariyawansa HA Bhat J Buyck B Cai L Dai YC Abd‐Elsalam KA Ertz D Hidayat I Jeewon R Jones EBG Bahkali AH Karunarathna SC Liu JK Luangsa‐ard JJ Lumbsch HT Maharachchikumbura SSN McKenzie EHC Moncalvo JM Ghobad‐Nejhad M Nilsson H Pang KL Pereira OL Phillips AJL Raspé O Rollins AW Romero AI Etayo J Selçuk F Stephenson SL Suetrong S Taylor JE Tsui CKM Vizzini A Abdel‐Wahab MA Wen TC Boonmee S Dai DQ Daranagama DA Dissanayake AJ Ekanayaka AH Fryar SC Hongsanan S Jayawardena RS Li WJ Perera RH Phookamsak R de Silva NI Thambugala KM Tian Q Wijayawardene NN Zhao RL Zhao Q Kang JC Promputtha I (2015) The Faces of fungi database: Fungal names linked with morphology, phylogeny and human impacts. Fungal Diversity 74(1): 3–18. 10.1007/s13225-015-0351-8

[B25] Jeewon R Hyde KD (2016) Establishing species boundaries and new taxa among fungi: Recommendations to resolve taxonomic ambiguities. Mycosphere 7(11): 1669–1677. 10.5943/mycosphere/7/11/4

[B26] Kearse M Moir R Wilson A Stones‐Havas S Cheung M Sturrock S Buxton S Cooper A Markowitz S Duran C Thierer T Ashton B Meintjes P Drummond A (2012) Geneious Basic: An integrated and extendable desktop software platform for the organization and analysis of sequence data. Bioinformatics (Oxford, England) 28(12): 1647–1649. 10.1093/bioinformatics/bts199 22543367 PMC3371832

[B27] Laurence WVA (2013) Isolation and Characterization of Endophytes Isolated from Akar Gaharu. Bachelor’s Research Dissertation, Universiti Malaysia Sarawak, Sarawak, Malaysia.

[B28] Lee SY Mohamed R (2016) The origin and domestication of *Aquilaria*, an important agarwood-producing genus. In: Mohamed R (Ed.) Chapter 1.Agarwood. Tropical Forestry. Springer, Berlin, Singapore, 1–20. 10.1007/978-981-10-0833-7_1

[B29] Li W Chen HQ Wang H Mei WL Dai HF (2021) Natural products in agarwood and *Aquilaria* plants: Chemistry, biological activities and biosynthesis. Natural Product Reports 38(3): 528–565. 10.1039/D0NP00042F 32990292

[B30] Li X Qu Z Zhang Y Ge Y Sun H (2022) Soil fungal community and potential function in different forest ecosystems. Diversity 14(7): 520. 10.3390/d14070520

[B31] Liao W Zou D Huang H Luo J Zhao C Wu Y (2018) Isolation and identification for pathogen of anthracnose in *Aquilariasinensis* (Lour.) Gilg. Nanfang Nongye Xuebao 49: 74–78.

[B32] Liew ECY Aptroot A Hyde KD (2000) Phylogenetic significance of the pseudoparaphyses in Loculoascomycete taxonomy. Molecular Phylogenetics and Evolution 16(3): 392–402. 10.1006/mpev.2000.0801 10991792

[B33] Liu YJ Whelen S Hall BD (1999) Phylogenetic relationships among ascomycetes: Evidence from an RNA polymerse II subunit. Molecular Biology and Evolution 16(12): 1799–1808. 10.1093/oxfordjournals.molbev.a026092 10605121

[B34] Liu YY Chen HQ Yang Y Zhang Z Wei J Meng H Chen WP Feng JD Gan BC Chen XY Gao ZH Huang JQ Chen B Chen HJ (2013) Whole-tree agarwood-inducing technique: An efficient novel technique for producing high-quality agarwood in cultivated *Aquilariasinensis* trees. Molecules (Basel, Switzerland) 18(3): 3086–3106. 10.3390/molecules18033086 23470337 PMC6270329

[B35] Maharachchikumbura SSN Chen Y Ariyawansa HA Hyde KD Haelewaters D Perera RH Samarakoon MC Wanasinghe DN Bustamante DE Liu J Lawrence DP Cheewangkoon R Stadler M (2021) Integrative approaches for species delimitation in Ascomycota. Fungal Diversity 109(1): 155–179. 10.1007/s13225-021-00486-6

[B36] Manawasinghe IS Hyde KD Wanasinghe DN Karunarathna SC Maharachchikumbura SSN Samarakoon MC Voglmayr H Pang KL Chiang MWL Jones EBG Saxena RK Kumar A Rajeshkumar KC Selbmann L Coleine C Hu Y Ainsworth AM Liimatainen K Niskanen T Ralaiveloarisoa A Arumugam E Kezo K Kaliyaperumal M Gunaseelan S Dissanayake AJ Khalid AN Gajanayake AJ Flakus A Armand A Aptroot A Rodrigues A Tsurykau A López-Villalba Á de Farias ARG Sánchez A Góes-Neto A Goto BT de Souza CAF Chuaseeharonnachai C Lin CG Li C Denchev CM Guerra-Mateo D Tennakoon DS Wei D-P Begerow D Alves E Drechsler-Santos ER Sousa ES de Medeiros EV Langer E Zhang F de Souza FA Magurno F Barreto GG Moreno G Mane G Alves-Silva G da Silva GA Xia G Shen H-W Gui H Senanayake IC Luangsa-ard JJ Liu JW Liu JK Ma J Lin JY Beserra Jr JEA Cano-Lira JF Gené J Harikrishnan K Lu L dos Santos LA Xu L Lacerda LT Gusmão LFP Cáceres MES Câmara MPS de Barros-Barreto MBB Calabon MS Kukwa M Kemler M de Melo MP Ghobad-Nejhad M Luo M Ding M Doilom M Phonemany M Usman M Thongklang N Boonyuen N Ashtekar N Kularathnage ND Sruthi OP Kwantong P Ansil PA Kooij PW Zhao Q Alfenas RF de Oliveira RJV Singh R da Silva RMF Avchar R Morey R Sharma R Xu RJ da Silveira RMB Xu RF Jayawardena RS Nanu S Nuankaew S Tibpromma S Boonmie S Somrithipol S Varghese S Moreira SI Rajwar S He SC Kumar TKA Denchev TT Luangharn T de Oliveira TGL Du TY Wen TC Du T Wu T Sri-Indrasutdhi V Doyle VP Baulin V Dong W Li WL Lu WH Tian W dos Vieira WA von Brackel W Yu XD Zhang X Liu XF Peng XC Chen Y Yang Y Gao Y Xiong Y Shu Y Lu YZ Shen YM Zhou Y Zhang YX Zhang W Luo ZL Madushani MA Cheewangkoon R Song JG Xu B (2024) Fungal diversity notes 1818–1918: taxonomic and phylogenetic contributions on genera and species of fungi. Fungal Diversity 1–261. 10.1007/s13225-024-00541-y

[B37] Mapook A Hyde KD McKenzie EHC Jones EBG Bhat DJ Jeewon R Stadler M Samarakoon MC Malaithong M Tanunchai B Buscot F Wubet T Purahong W (2020) Taxonomic and phylogenetic contributions to fungi associated with the invasive weed *Chromolaenaodorata* (Siam weed). Fungal Diversity 101(1): 175. 10.1007/s13225-020-00444-8

[B38] Miller MA Pfeiffer W Schwartz T (2010) Creating the CIPRES Science Gateway for inference of large phylogenetic trees. 2010 Gateway Computing Environments Workshop (GCE). IEEE Computer Society, New Orleans, LA, 1–8. 10.1109/GCE.2010.5676129

[B39] National Pharmacopoeia Committee (2020) Pharmacopoeia of the People’s Republic of China 2020 Version. Beijing. Chinese Medical Science and Technology Press 1: 192–193.

[B40] Niego AGT Lambert C Mortimer P Thongklang N Rapior S Grosse M Schrey H Charria-Girón E Walker A Hyde KD Stadler M (2023a) The contribution of fungi to the global economy. Fungal Diversity 121(1): 95–137. 10.1007/s13225-023-00520-9

[B41] Niego AGT Rapior S Thongklang N Raspé O Hyde KD Mortimer P (2023b) Reviewing the contributions of macrofungi to forest ecosystem processes and services. Fungal Biology Reviews 44: 100294. 10.1016/j.fbr.2022.11.002

[B42] Nizamani MM Zhang Q Zhang HL Wang Y (2023) Checklist of the fungi associated with the rubber tree (*Heveabrasiliensis*). Current Research in Environmental & Applied Mycology 13(1): 439–488. 10.5943/cream/13/1/17

[B43] Nylander JA Wilgenbusch JC Warren DL Swofford DL (2008) AWTY (are we there yet?): A system for graphical exploration of MCMC convergence in Bayesian phylogenetics. Bioinformatics (Oxford, England) 24(4): 581–583. 10.1093/bioinformatics/btm388 17766271

[B44] Punithalingam E Gibson IAS (1978) *Phomopsis* species from *Aquilariaagallocha*. Nova Hedwigia 29: 251–255.

[B45] Rambaut A (2012) FigTree version 1. 4. University of Edinburgh, Edinburgh.

[B46] Rasool S Mohamed R (2016) Understanding agarwood formation and its challenges. In: Mohamed R (Ed.) Agarwood tropical forestry.Springer, Berlin, 39–56. 10.1007/978-981-10-0833-7_3

[B47] Rathnayaka AR Tennakoon DS Jones GE Wanasinghe DN Bhat DJ Priyashantha AKH Stephenson SL Tibpromma S Karunarathna SC (2024) Significance of precise documentation of hosts and geospatial data of fungal collections, with an emphasis on plant-associated fungi. New Zealand Journal of Botany: 1–28. 10.1080/0028825X.2024.2381734

[B48] Rehner S (2001) Primers for elongation Factor 1‐Alpha (EF1‐Alpha). Insect Biocontrol Laboratory, USDA, ARS, PSI, Beltsville, MD.

[B49] Ronquist F Teslenko M Van Der Mark P Ayres DL Darling A Höhna S Larget B Liu L Suchard MA Huelsenbeck JP (2012) MrBayes 3.2: Efficient Bayesian phylogenetic inference and model choice across a large model space. Systematic Biology 61(3): 539–542. 10.1093/sysbio/sys029 22357727 PMC3329765

[B50] Senanayake IC Rathnayaka AR Marasinghe DS Calabon MS Gentekaki E Lee HB Hurdeal VG Pem D Dissanayake LS Wijesinghe SN Bundhun D Nguyen TT Goonasekara ID Abeywickrama PD Bhunjun CS Jayawardena RS Wanasinghe DN Jeewon R Bhat DJ Xiang MM (2020) Morphological approaches in studying fungi, collection examination isolation sporulation and preservation. Mycosphere 11(1): 2678–2754. 10.5943/mycosphere/11/1/20

[B51] Senwanna C Mapook A Samarakoon MC Karunarathna A Wang Y Tang AMC Haituk S Suwannarach N Hyde KD Cheewangkoon R (2021) Ascomycetes on Para rubber (*Heveabrasiliensis*). Mycosphere 12(1): 1334–1512. 10.5943/mycosphere/12/1/18

[B52] Šnajdr J Dobiášová P Větrovský T Valášková V Alawi A Boddy L Baldrian P (2011) Saprotrophic basidiomycete mycelia and their interspecific interactions affect the spatial distribution of extracellular enzymes in soil. FEMS Microbiology Ecology 78(1): 80–90. 10.1111/j.1574-6941.2011.01123.x 21539585

[B53] Stamatakis A (2014) RAxML version 8, a tool for phylogenetic analysis and post‐analysis of large phylogenies. Bioinformatics (Oxford, England) 30(9): 1312–1313. 10.1093/bioinformatics/btu033 24451623 PMC3998144

[B54] Stamatakis A Hoover P Rougemont J (2008) A rapid bootstrap algorithm for the RAxML web servers. Systematic Biology 57(5): 758–771. 10.1080/10635150802429642 18853362

[B55] Subansenee W Tongjiem N Sakekul V (1985) Fungi on agarwood [*Aquilaria* spp.]. Forest Product Research Division, Bangkok, 8. https://agris.fao.org/search/en/providers/122623/records/6471c0cf77fd37171a6e9504

[B56] Subasinghe SMCUP Hitihamu HID Fernando KMEP (2019) Use of two fungal species to induce agarwood resin formation in *Gyrinopswalla*. Journal of Forestry Research 30(2): 721–726. 10.1007/s11676-018-0654-1

[B57] Tao QG Du TY Zhang X Zhang Y Tian XG (2024) A new record of *Melomastiaclematidis* associated with *Aquilaria* sp. from Yunnan, China. MycoKing 3(2): 1–14.

[B58] Tedersoo L Bahram M Põlme S Kõljalg U Yorou NS Wijesundera R Ruiz LV Vasco-Palacios AM Thu PQ Suija A Smith ME Sharp C Saluveer E Saitta A Rosas M Riit T Ratkowsky D Pritsch K Põldmaa K Piepenbring M Phosri C Peterson M Parts K Pärte K Otsing E Nouhra E Njouonkou AL Nilsson RH Morgado LN Mayor J May TW Majuakim L Lodge DJ Lee SS Larsson K-H Kohout P Hosaka K Hiiesalu I Henkel TW Harend H Guo L-D Greslebin A Grelet G Geml J Gates G Dunstan W Dunk C Drenkhan R Dearnaley J Kesel AD Dang T Chen X Buegger F Brearley FQ Bonito G Anslan S Abell S Abarenkov K (2014) Global diversity and geography of soil fungi. Science 346(6213): 1256688. 10.1126/science.1256688 25430773

[B59] Tian XG Bao DF Karunarathna SC Jayawardena RS Hyde KD Bhat DJ Luo ZL Elgorban AM Hongsanan S Rajeshkumar KC Maharachchikumbura SSN Suwannarach N Dawoud TM Lu YZ Han JJ Xiao YP Du TY Lu L Xu RF Dai DQ Liu XF Liu C Tibpromma S (2024) Taxonomy and phylogeny of ascomycetes associated with selected economically important monocotyledons in China and Thailand. Mycosphere 15(1): 1–274. 10.5943/mycosphere/15/1/1

[B60] Tibpromma S Hyde KD McKenzie EHC Bhat JD Phillips AJL Wanasinghe DN Samarakoon MC Jayawardena RS Dissanayake AJ Tennakoon DS Doilom M Phookamsak R Tang AMC Xu JC Mortimer PE Promputtha I Maharachchikumbura SSN Khan S Karunarathna SC (2018) Fungal Diversity notes 840–928: Micro-fungi associated with Pandanaceae. Fungal Diversity 93(1): 1–160. 10.1007/s13225-018-0408-6

[B61] Tibpromma S Zhang L Karunarathna SC Du TY Phukhamsakda C Rachakunta M Suwannarach N Xu JC Mortimer PE Wang YH (2021) Volatile constituents of endophytic fungi isolated from *Aquilariasinensis* with descriptions of two new species of *Nemania*. Life (Basel, Switzerland) 11(4): 363. 10.3390/life11040363 PMC8073270 33921887

[B62] Vilgalys R Hester M (1990) Rapid genetic identification and mapping of enzymatically amplified ribosomal DNA from several *Cryptococcus* species. Journal of Bacteriology 172(8): 4238–4246. 10.1128/jb.172.8.4238-4246.1990 2376561 PMC213247

[B63] Wang S Yu Z Wang C Wu C Guo P Wei J (2018) Chemical constituents and pharmacological activity of agarwood and *Aquilaria* plants. Molecules (Basel, Switzerland) 23(2): 342. 10.3390/molecules23020342 29414842 PMC6017114

[B64] Wang Y Lin S Zhao L Sun X He W Zhang Y Dai YC (2019) *Lasiodiplodia* spp. associated with *Aquilariacrassna* in Laos. Mycological Progress 18(5): 683–701. 10.1007/s11557-019-01481-7

[B65] White TJ Bruns T Lee SJWT Taylor JL (1990) Amplification and direct sequencing of fungal ribosomal RNA genes for phylogenetics. In: Innis MA Gelfand DH Sninsky JJ White TJ (Eds) PCR protocols, a guide to methods and applications.Academic Press, San Diego, CA, 315–322. 10.1016/B978-0-12-372180-8.50042-1

[B66] Wijayawardene NN Hyde KD Dai DQ Sánchez-García M Goto BT Saxena RK Erdoğdu M Selçuk F Rajeshkumar KC Aptroot A Błaszkowski J Boonyuen N da Silva GA de Souza FA Dong W Ertz D Haelewaters D Jones EBG Karunarathna SC Kirk PM Kukwa M Kumla J Leontyev DV Lumbsch HT Maharachchikumbura SSN Marguno F Martínez-Rodríguez P Mešić A Monteiro JS Oehl F Pawłowska J Pem D Pfliegler WP Phillips AJL Pošta A He MQ Li JX Raza M Sruthi OP Suetrong S Suwannarach N Tedersoo L Thiyagaraja V Tibpromma S Tkalčec Z Tokarev YS Wanasinghe DN Wijesundara DSA Wimalaseana SDMK Madrid H Zhang GQ Gao Y Sánchez-Castro I Tang LZ Stadler M Yurkov A Thines M (2022) Outline of Fungi and fungus-like taxa – 2021. Mycosphere 13(1): 53–453. 10.5943/mycosphere/13/1/2

[B67] Xu YH Liao YC Zhang Z Liu J Sun PW Gao ZH Sui C Wei JH (2016) Jasmonic acid is a crucial signal transducer in heat shock induced sesquiterpene formation in *Aquilariasinensis.* Scientific Reports 6(1): 21843. 10.1038/srep21843 PMC4763180 26902148

[B68] Xu RF Karunarathna SC Phukhamsakda C Dai DQ Elgorban AM Suwannarach N Kumla J Wang XY Tibpromma S (2024) Four new species of Dothideomycetes (Ascomycota) from Pará Rubber (*Heveabrasiliensis*) in Yunnan Province, China. MycoKeys 103: 71–95. 10.3897/mycokeys.103.117580 38560534 PMC10980880

[B69] Yang EF Karunarathna SC Dai DQ Stephenson SL Elgorban AM Al-Rejaie S Xiong XR Promputtha I Samarakoon MC Tibpromma S (2022) Taxonomy and phylogeny of fungi associated with *Mangiferaindica* from Yunnan, China. Journal of Fungi (Basel, Switzerland) 8(12): 1249. 10.3390/jof8121249 36547582 PMC9780836

[B70] Zeng XY Tan TJ Tian FH Wang Y Wen TC (2023) OFPT: A one-stop software for fungal phylogeny. Mycosphere 14(1): 1730–1741. 10.5943/mycosphere/14/1/20

[B71] Zhang Y Du TY Han LS Tao QG Tian XG (2024) A new host record of *Allocryptovalsacastaneae* (Diatrypaceae) in *Aquilaria* from China. MycoKing 3(3): 1–15.

